# Preliminary study of Malaysian fruit bats species diversity in Lenggong Livestock Breeding Center, Perak: Potential risk of spill over infection

**DOI:** 10.14202/vetworld.2017.1297-1300

**Published:** 2017-11-02

**Authors:** Muhammed Mikail, T. A. Tengku Rinalfi Putra, Arshad Siti Suri, Mohd Noor Mohd Hezmee, M. T. Marina

**Affiliations:** 1 Research Centre for Wildlife, Faculty of Veterinary Medicine, Universiti Putra Malaysia, 43400, Serdang Selangor Darul Ehsan, Malaysia; 2 Department of Veterinary Pre Clinical Science, Faculty of Veterinary Medicine, Universiti Putra Malaysia, 43400, Serdang Selangor Darul Ehsan, Malaysia; 3 Directorate of Veterinary Services, Ministry of Agriculture and Natural Resources, Abubakar Umar Secretariat Bauchi, PMB 0068 Bauchi State, Nigeria; 4 Department of Veterinary Pathology and Microbiology, Faculty of Veterinary Medicine, Universiti Putra Malaysia, 43400, Serdang Selangor Darul Ehsan, Malaysia; 5 Centre of Foundation for Agricultural Studies, Universiti Putra Malaysia, 43400, Serdang Selangor Darul Ehsan, Malaysia

**Keywords:** Fruit bats, Lyssaviruses, Nipah virus, Wildlife sanctuaries, Zoonotic diseases

## Abstract

**Aim:**

Farms that are neighboring wildlife sanctuaries are at risk of spillover infection from wildlife, and the objective of this research is to examine the species diversity of Malaysian fruit bats in livestock farm in determining the possible risk of spill over infection to livestock.

**Materials and Methods:**

Fifty individual fruit bats were captured using six mists net, from May to July 2017. The nets were set at dusk (1830 h) as bats emerge for foraging and monitored at every 30-min intervals throughout the night until dawn when they returned to the roost. The nets were closed for the day until next night, and captured bats were identified to species levels.

**Results:**

All the captured bats were mega chiropterans, and *Cynopterus brachyotis* was the highest captured species, representing 40% of the total capture. Shannon–Weiner index is 2.80, and Simpson index is 0.2. Our result suggests that there is a degree of species dominance with low diversity in Lenggong Livestock Breeding Center.

**Conclusion:**

We concluded that fruit bats are indeed, encroaching livestock areas and the species identified could be a potential source of infection to susceptible livestock. Hence, an active surveillance should be embarked on farms that border wildlife sanctuaries.

## Introduction

Bats of the order Chiroptera are classified into two suborders: Yinpterochiroptera (Megachiropterans/Megabats) and Yangochiroptera (Microchiroptera/Microbats) [1]. They are the single largest in species richness among mammals with more than 1300 species currently recognized and distributed widely except in Antarctica [2]. Bats are recognized as a reservoir host of many viruses which are zoonotic in nature and exhibit a co-evolutionary relationship with many zoonotic viruses [3,4]. Despite their importance in zoonotic disease transmission, they also play an important role in biotic community such as in insect control, pollination, and seed dispersal [2]. Luis *et al*. [5] reported that bats are unique from all other mammals due to their specific morphological and physiological adaptations for powered flight and ecosystem services. Their significant link with human diseases particularly zoonotic viruses is of great concern. Moreover, there are about 110 species of bats in Peninsular Malaysia, out of which 105 have a known locality records while 5 lacks or do not have a precise locality [6]. Bats in Peninsular Malaysia are threatened with habitat loss due to the conversion of forest into agricultural and urban areas [7].

This study was undertaken to determine the possibilities of fruit bats encroaching livestock farm that borders wildlife sanctuary and determine the species diversity of fruit bats in Lenggong livestock breeding center for potential spill over infection.

## Materials and Methods

### Ethical approval

This research was approved by the Institutional Animal Care and Use Committee Universiti Putra Malaysia, UPM/IACUC/AUP-R094/2016.

### Study site

**Figure-1:**
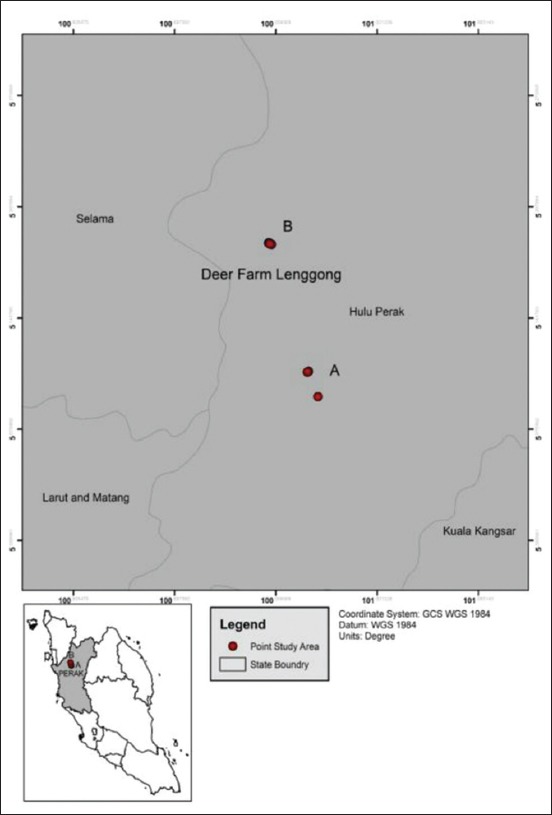
Map of Lenggong Livestock Breeding Center.

This study was conducted in Lenggong Livestock Breeding Centre (LLBC) which is located in Hulu Perak Lenggong, a town in Perak Malaysia, about 75 km North of Ipoh town located at 100º 956315’ Eand 5º 184752’ N (Figure-1) It is a deer farm owned by the Department of Veterinary Services, with 821 deers consisting of Timoreses, Samba and Axis. The deers are kept in semi-wild environment in multiple paddocks of about 1 acre in size.

### Bats sampling

**Figure-2:**
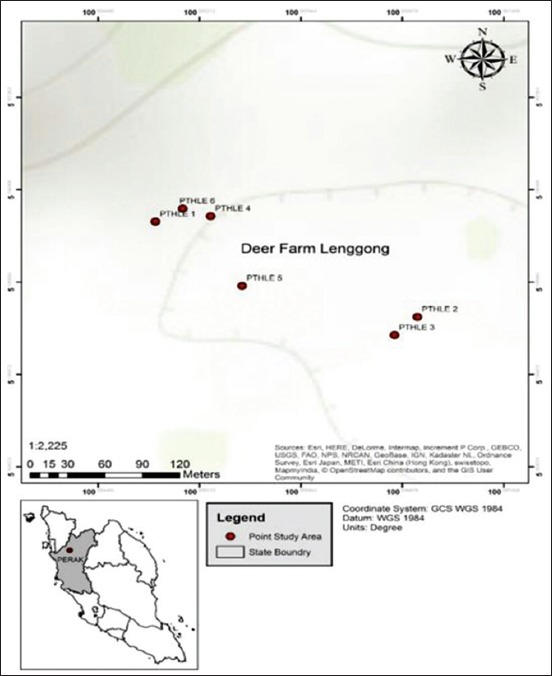
Map of sampling points.

Five sampling plots were selected randomly, within the peripheral areas of the farm, which has some fruit trees such as star fruit, rambutan, and ciku tree (Figure-2). Sampling plots were selected based on their possibilities as roosting sites for fruit bats and expected flying paths of the bats. Mist nets (9 m×4 m) black, made up of Nylon with a mesh size of 2.5 mm, 4 shelves, and length of 9 m, supported with two aluminum poles of about 4 m, was set and erected in each sampling plot. The nets were set at dusk (1830 h) as bats emerge for foraging and monitored at every 30-min intervals throughout the night until dawn when they returned from the roost, and then, nets were closed for the day until next night. The captured bats were removed from the net with a gloved hand and transferred into a cotton cloth bag. Bats were grasp in the palm of the hand and the fingers curled around the body with the head between the thumb and first finger for measurements and identifications as described by Kingston *et al.*, [8].

Data were analyzed using Shannon–Weiner diversity index, evenness, species richness, Simpson index, and true effective number of species, and relative abundance was also determined.

## Result

A total of 50 individual bats from family Pteropodiformes were captured during the 3 months period of sampling (Table-1). The species include *Cynopterus brachyotis, Cynopterus horsfieldii, Eonycteris spelaea, Macroglossus sobrinus* and *Megaerops ecaudatus*, respectively. However, *C. brachyotis* or the lesser short-nosed fruit bat was the most frequently captured bat during the sampling period with a total of 20 bats. The high rate of bat capture was recorded in July, in which 27 capture was recorded, followed by May with 15 i and the least capture was in June with 8 bats. Mist net set near to fruiting trees recorded the highest capture rate. A total of 20 females and 30 males were captured, out of which 47 were adults and only 3 were young (Table-2). Cynopterus *brachyotis* has the highest relative abundance (40%), and *M. ecaudatus* has the lowest relative abundance (2%). Shannon–Weiner index for this study is 2.80 while evenness is 1.75. Simpson index is 0.2 with an index of diversity of 0.8. The effective number of species for Shannon–Weiner index is 16.44 while for Simpson index is 1.25.

**Table-1:** Bat species. captured during the period of May-July 2017 at Lenggong Deer Farm.

Name of species	No of individuals	Male	Female	Relative abundance (n/Nx100)
*Cynopterus brachyotis*	20	12	8	40
*Cynopterus horsfieldii*	13	7	6	26
*Eonycteris spelaea*	14	9	5	28
*Macroglossus sobrinus*	2	2	0	4
*Megaerops ecaudatus*	1	0	1	2
Total (N)	50	30	20	

**Table-2:** Summary of the result.

Species richness	5
Shannon–Weiner index	2.80
Evenness	1.75
Simpson index	0.2
Simpson index of diversity	0.8
Shannon effective number	16.44
Simpson effective number	1.25

## Discussion

Fifty individual fruit bats were captured during the preliminary sampling period from May- July. The captured bats are representative of a small portion of Malaysian fruit bats; however, the rate of bat capture in LLBC was low, when compared with other studies, as reported in related studies at Kuala Atok, within the period of 7 days of sampling, a total of 79 individual bats were captured in Kuala Atok, Taman Negara Pahang [9]. The result shows a low diversity of fruit bats in livestock farm, and the Simpson effective value of the study is less than the Shannon effective value, which implies a degree of fruit bat dominance in the farm. Fruit bats dominate LLBC as a result of available fruit trees in the farms such as the rambutan, star fruit, and ciku trees which are potential roosting and foraging areas of Pteropodiformes [10]. The highest capture rate of *Cynopterus brachyotis* agreed with other previous findings [9]. *Cynopterus brachyotis* and *Cynopterus horsfieldii* are the most common and abundant species in Peninsular Malaysia and are known to occupy all types of habitat such as lowland, hills, sub-montane, montane and mangrove forest, orchards, and plantations [9,11]. Sampling in July had the highest capture due to non-rainy nights compared to May.

### Possible risk of spillover infection from wildlife to susceptible livestock

Following an outbreak of Nipah virus infection in Peninsular Malaysia, active wildlife surveillance was embarked on the fruit bats and species such as *C. brachyotis* and *E. spelaea* which they shows evidence of neutralizing antibodies to Nipah virus with antibody prevalence rate of 4% and 5%, respectively [12]. Nipah virus was confirmed to infect pigs in a farm following consumption of contaminated partially eaten fruit of bats, and then, transmission between pigs to pigs and subsequently to human that comes in contact with infected pig [13].

Livestock in LLBC are allowed to roam freely in semi-wild environment. This poses a great risk similar to the transmission pathways of Nipah virus in which contact with contaminated fruit or access to water contaminated with infected bat saliva can be difficult to prevent in LLBC. It is previously known that drinking raw contaminated date palm sap transmits Nipah virus infections to humans [14]. Lyssavirus belongs to the family Rhabdoviridae, currently, 14 lyssaviruses were recognized, and all lyssaviruses with the exception of Mokola Virus are known to spillover from bats to susceptible wildlife, livestock/pet, and humans through the saliva following bites as the common transmission pathways [15,16]. In Asia and Southeast Asia, the only detectable bat lyssavirus is Australian bat lyssavirus and Gannoruwa bat lyssavirus [15,17]. However, *Cynopterus* sphinx has been reported to demonstrate rabies virus neutralizing antibodies in India [18], and contact with wildlife reservoir host of rabies, in Africa, had resulted to spillover infection to susceptible African civet in Serengeti National Park Tanzania [19]. *Cynopterus* species is a common fruit bat of Peninsular Malaysia and was reported in our study with high capture rate, and preliminary nucleic acid detection shows a positive result of bat lyssaviruses in these species (*Cynopterus. brachyotis* and *Cynopterus horsfieldii)*.

## Conclusion

This study, the first of it is kind in Malaysia, reports bats encroaching livestock farm that borders wildlife sanctuary and the species identified could be a potential source of infection to susceptible livestock. The findings would act as a guide for control and prevention policies of a possible outbreak.

## Recommendations

Active wildlife surveillance in livestock farm should be embarked on farms bordering wildlife sanctuaries and forest reserves, with the view of detecting wildlife reservoir host. Subsequent work should include the ecology of zoonotic diseases of fruit bats origin.

## Authors’ Contributions

MM, TATRP, MNMH, ASS and MMT collectively designed, carried out the sampling and species identification. TRPTA, MNMH, MMT, and ASS reviewed the manuscript, and TATRP approved the final manuscript. All authors read and approved the final manuscript.
